# FLT3-ITD Mutation and Allogeneic Stem Cell Transplantation in Acute Myeloid Leukemia: A Case Study

**DOI:** 10.7759/cureus.42504

**Published:** 2023-07-26

**Authors:** Shahzeb Saeed, Raghu Halappa Nagaraj, Han Grezenko, Abdur Rehman, Abdullah Shehryar, Mohammad Ahsan Anwaar, Slobodan Lazarevic, S. M. Iram Shahzed, Archana Das, Karla I Vargas

**Affiliations:** 1 Internal Medicine, Army Medical College, Rawalpindi, PAK; 2 Surgery, Avalon University School of Medicine, Willemstad, CUW; 3 Translational Neurosciences, Barrow Neurological Institute, Phoenix, USA; 4 Surgery, Mayo Hospital, Lahore, PAK; 5 Internal Medicine, Allama Iqbal Medical College, Lahore, PAK; 6 Internal Medicine, CMH (Combined Military Hospital) Lahore Medical College and Institute of Dentistry, Lahore, PAK; 7 Internal Medicine, Faculty of Medicine, University of Niš, Niš, SRB; 8 Internal Medicine, South Brooklyn Health, Brooklyn, USA; 9 Internal Medicine, North East Medical College and Hospital, Sylhet, BGD; 10 Medicine, Universidad Juárez del Estado de Durango, Durango, MEX

**Keywords:** hematologic cancer, molecular profiling risk stratification, molecular profiling, allogeneic stem cell transplantation, flt3-itd mutation, acute myeloid leukemia (aml)

## Abstract

Acute myeloid leukemia (AML) is a hematologic cancer that is characterized by unchecked myeloid precursor cell growth in the bone marrow and peripheral circulation, which results in an overabundance of immature myeloid cells. The 22-year-old man featured in this case report had a fever, tiredness, and easy bruising. Pancytopenia was discovered through laboratory testing, and an AML diagnosis was confirmed by a bone marrow biopsy, with myeloid blasts making up 85% of the nucleated cells. FLT3-ITD and NPM1 mutations were found by genetic testing. After receiving induction chemotherapy using the drugs daunorubicin and cytarabine, the patient experienced complete remission after just one cycle of treatment. He then had an allogeneic stem cell transplant and was still in remission during follow-up. This example highlights the significance of early AML diagnosis and detection, as well as the function of molecular profiling and risk stratification in directing treatment choices. It emphasizes the requirement for continued study to produce novel treatments and enhance results for AML patients. In general, this case study advances knowledge of AML and its management techniques. For AML patients to experience the best results, early diagnosis, risk assessment, and individualized therapy plans based on molecular profiling are essential. AML patients' prognosis and quality of life can be improved by the development of targeted medicines, which require ongoing study to better understand the disease.

## Introduction

Acute myeloid leukemia (AML) is a hematological malignancy characterized by the uncontrolled proliferation of myeloid precursor cells in the bone marrow. AML is a relatively rare disease, accounting for approximately 1.2% of all new cancer cases in the United States [1]. It is most commonly diagnosed in older adults, with a median age at diagnosis of 67 years [1]. However, AML can also occur in young adults and children, albeit less frequently.

AML arises from genetic mutations that cause abnormal differentiation and proliferation of myeloid precursor cells. The genetic abnormalities in AML can involve various genes and pathways, including FLT3, NPM1, CEBPA, and RUNX1 [2]. The classification of AML is based on the World Health Organization (WHO) criteria, which incorporate morphological, cytogenetic, and molecular features of the disease. The WHO classification system divides AML into various subtypes, including de novo AML, therapy-related AML, and AML with myelodysplasia-related change [3].

The clinical presentation of AML is highly variable and depends on the degree of bone marrow infiltration and cytopenias. The most common presenting symptoms of AML include fatigue, fever, and bleeding disorders such as easy bruising or petechiae. Patients may also present with infections or constitutional symptoms, such as weight loss and night sweats [4]. The diagnosis of AML is based on the presence of ≥ 20% blasts in the bone marrow or peripheral blood.

The treatment of AML typically involves induction chemotherapy followed by consolidation therapy. The choice of chemotherapy regimen and the need for transplantation depend on the patient's age, performance status, cytogenetic and molecular risk factors, and response to induction therapy [5]. Allogeneic stem cell transplantation is considered the standard of care for eligible patients with intermediate-risk or high-risk AML, as it provides a potential cure for the disease. However, transplantation is associated with significant morbidity and mortality, and careful patient selection is necessary [5].

Despite recent advances in treatment, the prognosis of AML remains highly variable, with better outcomes observed in younger patients, those with favorable cytogenetics, and those who achieve complete remission [6].

## Case presentation

A 22-year-old male patient arrived at the emergency room complaining of fatigue, fever, and easy bruising for the previous two weeks. He reported feeling generally unwell and had noticed multiple small red spots on his arms and legs that had appeared spontaneously. He denied any recent illness, travel, or significant past medical history. He was not taking any medications and had no family history of hematological disorders.

On physical examination, the patient appeared pale and fatigued. He had multiple petechiae and ecchymoses on his arms and legs and scattered erythematous papules on his chest and back. There was no lymphadenopathy or organomegaly appreciated. The initial vital signs were stable, with a temperature of 37.5°C, heart rate of 90 beats per minute, blood pressure of 120/70 mmHg, and oxygen saturation of 98% on room air.

Laboratory testing revealed pancytopenia, with a white blood cell count of 1.5 x 10^9^/L, hemoglobin of 7.5 g/dL, and platelet count of 50 x 10^9^/L. The peripheral smear showed leukopenia with few neutrophils, anemia with normochromic normocytic red blood cells, and thrombocytopenia with decreased platelet count and large platelets. The coagulation profile was normal, and there were no signs of hemolysis. The serum chemistry panel and liver function tests were within normal limits.

**Figure 1:**
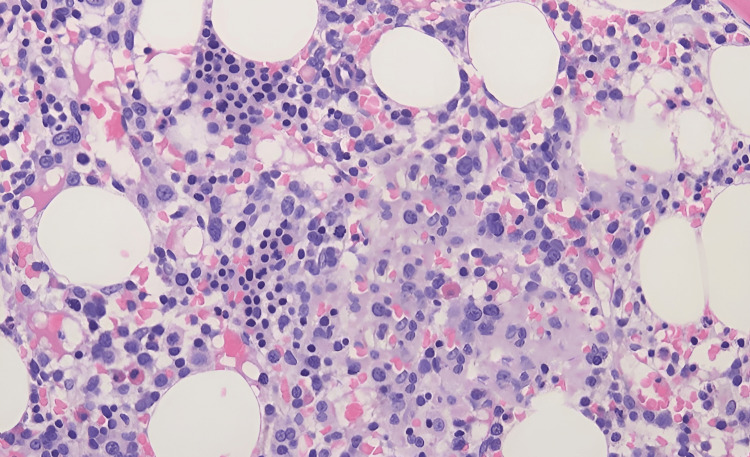
The bone marrow core biopsy shows a markedly hypercellular bone marrow with sheets of blasts.

Given the patient's presentation, a bone marrow biopsy was performed to investigate the underlying cause of pancytopenia. The biopsy revealed myeloid blasts comprising 85% of the nucleated cells, confirming the diagnosis of AML (Figure 1).

Cytogenetic studies were performed, which revealed a normal karyotype. Molecular analysis showed FLT3-ITD and NPM1 mutation, and the absence of FLT3-TKD mutation.

The patient was started on induction chemotherapy with daunorubicin and cytarabine, and he achieved complete remission after one cycle of therapy. The patient underwent allogeneic stem cell transplantation and remained in remission at the last follow-up visit.

## Discussion

AML is a malignant hematologic disorder characterized by the clonal proliferation of myeloid precursors, resulting in the accumulation of immature myeloid cells in the bone marrow and peripheral blood. It is the most common acute leukemia in adults, with an incidence of approximately three to four cases per 100,000 individuals per year in the United States [7]. The diagnosis of AML is based on the presence of at least 20% blasts in the bone marrow or peripheral blood, with evidence of dysplasia or maturation arrest in one or more myeloid lineages.

In this case, the patient presented with symptoms of fatigue, fever, easy bruising, pancytopenia, and petechiae on the skin. The bone marrow biopsy confirmed the diagnosis of AML with myeloid blasts comprising 85% of the nucleated cells. The cytogenetic and molecular analysis revealed FLT3-ITD mutation, NPM1 mutation, and absence of FLT3-TKD mutation, which are commonly seen in AML and are associated with poor prognosis.

The treatment of AML typically involves induction chemotherapy with cytarabine and an anthracycline, such as daunorubicin or idarubicin, followed by consolidation therapy with additional chemotherapy or allogeneic stem cell transplantation [8]. Induction therapy aims to achieve complete remission, defined as less than 5% blasts in the bone marrow and restoration of normal hematopoiesis. The risk stratification of AML is based on factors such as age, performance status, cytogenetics, and molecular profile, which help guide the choice of therapy and predict the likelihood of achieving remission and long-term survival [9].

In this case, the patient was treated with daunorubicin and cytarabine for induction therapy and achieved complete remission after one cycle of treatment. He subsequently underwent allogeneic stem cell transplantation, which is recommended for patients with high-risk features, such as FLT3-ITD mutation, and can provide a potential cure for AML. The patient remained in remission at the time of the last follow-up visit.

The molecular profiling of AML has revolutionized the diagnosis and management of this disease, allowing for risk stratification and the development of targeted therapies [10]. The FLT3 mutation, which is present in approximately 30% of AML cases, is associated with poor prognosis and resistance to chemotherapy. The development of FLT3 inhibitors, such as midostaurin and gilteritinib, has improved the outcomes for patients with FLT3-mutated AML, and these agents are now recommended for use in combination with chemotherapy or as monotherapy in specific settings [11].

## Conclusions

This case emphasizes the necessity of early detection and diagnosis of AML as well as the use of molecular profiling and risk stratification in determining the most appropriate course of treatment. For patients with high-risk characteristics like the FLT3-ITD mutation, improvements in therapy such as targeted medicines and allogeneic stem cell transplantation have improved results. Due to the possibility of recurrence, regular monitoring and follow-up are also essential. The best chance for obtaining remission and extending survival is early identification and rapid beginning of salvage therapy. A multidisciplinary strategy including numerous healthcare professionals such as hematologists/oncologists, radiation oncologists, bone marrow transplant specialists, and pathologists is necessary for effective AML management. Individualized treatment based on disease features and patient demands is ensured by collaborative care. This case emphasizes the ongoing need for research, development of new therapies, and clinical trials to enhance outcomes and quality of life for AML patients.
